# Growth hormone secretagogues modulate inflammation and fibrosis in *mdx* mouse model of Duchenne muscular dystrophy

**DOI:** 10.3389/fimmu.2023.1119888

**Published:** 2023-04-12

**Authors:** Brigida Boccanegra, Ornella Cappellari, Paola Mantuano, Daniela Trisciuzzi, Antonietta Mele, Lisamaura Tulimiero, Michela De Bellis, Santa Cirmi, Francesca Sanarica, Alessandro Giovanni Cerchiara, Elena Conte, Ramona Meanti, Laura Rizzi, Elena Bresciani, Severine Denoyelle, Jean-Alain Fehrentz, Gabriele Cruciani, Orazio Nicolotti, Antonella Liantonio, Antonio Torsello, Annamaria De Luca

**Affiliations:** ^1^ Department of Pharmacy – Drug Sciences, University of Bari “Aldo Moro”, Bari, Italy; ^2^ School of Medicine and Surgery, University of Milan-BICOCCA, Milan, Italy; ^3^ Institut des Biomolécules Max Mousseron, UMR 5247 CNRS-Université Montpellier-ENSCM, Faculté de Pharmacie, Montpellier, France; ^4^ Department of Chemistry, Biology and Biotechnology, University of Perugia, Perugia, Italy

**Keywords:** Duchenne muscular dystrophy, mdx mouse, growth hormone secretagogues, skeletal muscle, fibrosis

## Abstract

**Introduction:**

Growth hormone secretagogues (GHSs) exert multiple actions, being able to activate GHS-receptor 1a, control inflammation and metabolism, to enhance GH/insulin-like growth factor-1 (IGF-1)-mediated myogenesis, and to inhibit angiotensin-converting enzyme. These mechanisms are of interest for potentially targeting multiple steps of pathogenic cascade in Duchenne muscular dystrophy (DMD).

**Methods:**

Here, we aimed to provide preclinical evidence for potential benefits of GHSs in DMD, *via* a multidisciplinary *in vivo* and *ex vivo* comparison in *mdx* mice, of two *ad hoc* synthesized compounds (EP80317 and JMV2894), with a wide but different profile. 4-week-old *mdx* mice were treated for 8 weeks with EP80317 or JMV2894 (320 µg/kg/d, s.c.).

**Results:**

*In vivo*, both GHSs increased mice forelimb force (recovery score, RS towards WT: 20% for EP80317 and 32% for JMV2894 at week 8). In parallel, GHSs also reduced diaphragm (DIA) and gastrocnemius (GC) ultrasound echodensity, a fibrosis-related parameter (RS: ranging between 26% and 75%). *Ex vivo*, both drugs ameliorated DIA isometric force and calcium-related indices (*e.g.*, RS: 40% for tetanic force). Histological analysis highlighted a relevant reduction of fibrosis in GC and DIA muscles of treated mice, paralleled by a decrease in gene expression of *TGF-β1* and *Col1a1*. Also, decreased levels of pro-inflammatory genes (*IL-6, CD68*), accompanied by an increment in *Sirt-1, PGC-1α* and *MEF2c* expression, were observed in response to treatments, suggesting an overall improvement of myofiber metabolism. No detectable transcript levels of *GHS receptor-1a*, nor an increase of circulating IGF-1 were found, suggesting the presence of a novel receptor-independent mechanism in skeletal muscle. Preliminary docking studies revealed a potential binding capability of JMV2894 on metalloproteases involved in extracellular matrix remodeling and cytokine production, such as ADAMTS-5 and MMP-9, overactivated in DMD.

**Discussion:**

Our results support the interest of GHSs as modulators of pathology progression in *mdx* mice, disclosing a direct anti-fibrotic action that may prove beneficial to contrast pathological remodeling.

## 1 Introduction

Duchenne muscular dystrophy (DMD) is a severe inherited dystrophy of childhood, affecting 1 in 5,000 live male births due to X-linked mutations in the dystrophin gene that prevent the expression of functional dystrophin at the sarcolemma of individual muscle fibers (1). Dystrophin links the extracellular matrix (ECM) to the cytoskeleton of myofibers *via* the dystrophin-associated glycoprotein complex (DGC) (2). The absence of dystrophin results in permanent fragility and leakiness of the sarcolemma, which leads to increased calcium influx and progressive myofiber death, with subsequent loss of muscle tissue, ineffective regeneration, and progressive fibrosis (3). Strategies for gene correction and replacement have been actively pursued in the last decades; although existing hurdles, some drugs have been conditionally approved for paths of personalized medicine. To date, the standard of care comprises the early use of glucocorticoids, the sole drugs able to prolong ambulation and control symptoms. However, their use is controversial, due to the severe side effects, involving delay of growth, bone demineralization and metabolic dysfunctions (4). Then, the search of small drugs able to act as disease modifiers in all DMD patients, irrespective of the mutation, is an important goal to be achieved with a robust and proper preclinical investigation (5).

Several preclinical studies allowed a better understanding of the complex cascade of events leading to muscle wasting in dystrophin-deficient myofibers (6–8). The *mdx* mouse model, widely used, develops a less severe phenotype, but it proved to be useful as it recapitulates the main alterations of DMD patients. Dystrophin and DGC are active components of a mechano-transduction machinery allowing proper force transmission from contractile apparatus to extracellular matrix, meanwhile contributing to intracellular signaling in response to mechanical stressors (1, 9). Accordingly, dystrophic muscles are more susceptible to contraction-induced injury and are also unable to properly adapt muscle physiology, as well as their metabolism and antioxidant defenses in response to contractile stress (10–13). In these conditions, an impairment of AMPK-Sirt-1-PGC-1α pathway is observed and is likely responsible for a failing signaling of mitochondrial biogenesis and for an unbalance between damaging and protective signals that may account for uncontrolled chronic inflammation and oxidative stress. Indeed, this latter is an intrinsic and early sign of myofiber distress and contributes to pathology progression reinforcing various pathways, such as inflammation and aberrant calcium influx, *via* modulation of redox-sensitive targets (14, 15). One of the main sources of reactive oxygen species (ROS) is the NADPH-oxidase (NOX), whose expression and activity are increased in both skeletal muscle and heart of dystrophic subjects, in relation to microtubule dysfunction and mechanical challenge (9, 11). Among cellular mechanisms causing ROS production, those associated to angiotensin II (ANG II)-activated pathways are of particular interest. Indeed, ANG II, the main effector molecule of renin-angiotensin-aldosterone system (RAAS), exerts pro-oxidant, pro-inflammatory, and pro-fibrotic actions in both heart and skeletal muscle (16, 17). Importantly, ACE inhibitors are elective drugs in the management of cardiomyopathy and late heart failure of DMD patients, while preclinical studies in *mdx* mice underlined a prominent role of ANG II-related pathway in early inflammation and in late fibrosis in skeletal muscle (4, 18).

The auto-reinforcing loop associated to all reported mechanisms involved in DMD, drastically hampers the efficacy of highly selective drugs. Thus, the complexity of the pathology pushes to search for drugs acting upstream and downstream of the cascade to exert a wider protection. This objective could be achieved through combined therapy or by using drugs with multiple activities. This latter possibility is at the basis of our interest in growth hormone secretagogues (GHSs).

The enhancement of growth hormone (GH)/insulin-like growth factor-1 (IGF-1) axis by the direct use of GH or IGF-1 has been proposed in DMD to improve muscle anabolism and myogenic program and for reducing the deleterious effects of glucocorticoids on patients’ growth (4, 19). However, their clinical use is controversial due to side effects. Ghrelin and des-acyl ghrelin are 28 amino acid hormones produced primarily by the stomach, and acting through high affinity binding to the growth hormone secretagogues receptor type 1a (GHS-R1a), inducing GH release and promoting food intake, positive energy balance, and increase in lean body mass (20). Acylated and des-acyl ghrelin are also endowed with several extra-endocrine activities. In fact, both exhibit anti-inflammatory, antioxidative and antiapoptotic activities, and might represent an efficient approach to contrast fibrosis, likely *via* a mechanism involving inhibition of NOX-ROS signaling (21). Accordingly, ghrelin and des-acyl ghrelin protect muscle from atrophy and wasting in a wide variety of experimental models (22, 23).

Importantly, it has been demonstrated that genetically induced upregulation of circulating or local des-acyl ghrelin in *mdx* mice improves the dystrophic phenotype, including muscle architecture and functionality. In parallel, *in vitro* experiments on dystrophin-null satellite cells demonstrated that des-acyl ghrelin regulates multiple steps of muscle regeneration by stimulating asymmetric division-mediated satellite cells self-renewal and by promoting terminal differentiation and fusion of proliferating myoblasts (24).

Considering the limited clinical usefulness of ghrelin for its short half-life and low plasma stability (25), several synthetic GHSs have been developed for various diseases requiring a ghrelin mimetic activity and some of them are under evaluation by the European Medicine Agency for approval in cancer cachexia. Synthetic GHSs maintain a wide, and still under clarification, mechanism of action accounting for multifaceted actions. These include an increase in food intake, a preservation of lean body mass, a decrease in inflammation and a stimulation of GH release (26). Synthetic GHSs are able to contrast cisplatin induced cachexia, ameliorating calcium homeostasis while reducing oxidative stress and stimulating mitochondrial biogenesis in skeletal muscle (27–29). Additionally, hexarelin and several GHSs are short peptides and peptoids, with a similar structure to that of several ACE inhibitors and, for some of them, an effective inhibitory ACE activity has been described *in vitro* (30). The entire spectrum of actions reinforces the interest of GHSs in muscular dystrophy, although their therapeutic potential in DMD has not been preclinically assessed so far. Therefore, the main goal of the present work was to offer a sound preclinical basis for putative clinically relevant effects in DMD of two selected GHSs (EP80317 and JMV2894), with a wide profile. The two compounds were sub-chronically administered at the dose of 320 µg/kg/d to *mdx* mice for assessing their effects on disease relevant readouts by means of a multidisciplinary *in vivo/ex vivo* approach.

## 2 Materials and methods

### 2.1 *In vivo* studies

#### 2.1.1 Animal groups and treatments

All the experiments were conducted in conformity with the Italian Guidelines for Care and Use of Laboratory Animals (D.L.116/92) and with the European Directive (2010/63/UE) and in compliance with the ARRIVE guidelines. The study was approved by the National Ethics Committee for Research Animal Welfare of the Italian Ministry of Health (authorization no.166/2020-PR). Most of the experimental *in vivo* and *ex vivo* procedures followed international TREAT-NMD guidelines for pre-clinical studies in neuromuscular diseases (http://www.treat-nmd.eu/research/preclinical/SOPs/) (5).

Both 4-week-old male mice C57BL/10ScSn/J (wild type, WT) and C57BL/10ScSn*Dmd^mdx^*/J (*mdx*) were purchased from The Jackson Laboratory, USA (distributed by Charles River Laboratories, Calco, Italy) and allocated to the following experimental groups: a) WT (n = 7); b) *mdx* + vehicle (n = 10), *mdx* + EP80317 (n = 8), *mdx* + JMV2894 (n = 8). Tested compounds were synthesized and analyzed for purity in-house according to previously described process (31). Dystrophic mice were randomly assigned to the various groups. All mice were housed in suitable cages (max. 5 mice *per* cage) and acclimatized to local housing conditions (22 – 24°C, humidity 50 – 60%, and 12 h light/12 h dark cycle) for approximately 5 days. All mice were fed a daily amount of chow (VRF1 standard pelleted diet, Charles River Laboratories) of 5 g *per* mouse. This fixed amount, largely exceeding the physiological daily consumption (~ 3 g/mouse) allowed a proper control by the experimenter of any possible macroscopic deviation in food consumption between groups, which in fact did not occur. Filtered tap water was available *ad libitum*. Both drugs were administered s.c. for 8 weeks (T0 – T8), at the dose of 320 µg/kg/day, 6 d/w. All mice were non-invasively and longitudinally monitored for health, well-being throughout the total study period. Body mass (BM; g) variations were regularly assessed at the start of each experimental week.

#### 2.1.2 Forelimb force and hind limb plantar flexor torque

Forelimb grip strength was measured on a weekly basis by means of a grip strength meter (Columbus Instruments, USA) and according to a standard protocol [TREAT-NMD SOP (ID) Number: DMD_M.2.2.001]. Maximal force (expressed in kg force, KGF), obtained from five repeated measurements *per* mouse, was used for data analysis (12, 32).

Isometric torque of hind limb plantar flexors (gastrocnemius – GC, soleus – SOL, plantaris muscles) were measured at T8 in anesthetized mice *via* the 1300A 3-in-1 Whole Animal System (Aurora Scientific Inc., Aurora, ON, Canada). A series of isometric contractions, elicited *via* percutaneous electrical stimulation of the sciatic nerve, were recorded at increasing frequencies (1 – 200 Hz); data for plantar flexor torque obtained at each frequency were normalized to BM (N*mm/kg) and used to generate torque – frequency curves (12, 33–35).

#### 2.1.3 Hind limb and diaphragm ultrasonography

Ultrasonography was performed *in vivo* at T8 *via* an ultra-high frequency ultrasound biomicroscopy system (Vevo® 2100; VisualSonics, Toronto, ON, Canada). Each animal, properly anesthetized *via* inhalation (36–38), was placed on a thermostatically controlled table (37°C) equipped with four copper leads, allowing to monitor both heart and respiratory rate during the imaging session. Body temperature was constantly monitored *via* a rectal probe. Each hind limb of the animal was shaved, secured parallel to the body (foot at 90° with the limb), and covered with ultrasound gel. A three-dimensional (3D) volume scan was obtained by acquiring multiple two-dimensional (2D) images of the long hind limb axis at regular intervals in B-dimensional mode (B-mode) by using an MS250 probe (frequency of 21 MHz; lateral and axial resolutions of 165 and 75 μm, respectively). At the end of the procedure, 3D images were reconstructed from previously collected multiple 2D frames and visualized with VisualSonics 3D software, to obtain the total hind limb volume (in mm^3^) (36, 37). For each mouse 3 – 4 frames, in which gastrocnemius (GC) muscle was clearly visible, were chosen to evaluate echodensity measured using ImageJ^®^ software by creating a grey scale analysis histogram.

For diaphragm (DIA) imaging, both mouse and probe were properly positioned (38). The same high-resolution probe (MS250) was used to perform mono-dimensional (M- Mode) and B-Mode acquisitions. DIA movement amplitude was measured in M-Mode during normal breathing cycles on the left side, which provides less variability in measurements for all experimental groups. The amplitude during each inspiration (positive deflection) was measured as the distance (in mm) between the baseline and the peak of contraction. For each mouse, DIA amplitude was calculated as the mean value obtained from 3 – 5 measurements (38). Images acquired in B-Mode were used to evaluate DIA echodensity, which was measured using ImageJ^®^ software by creating a grey scale analysis histogram on the entire outlined DIA section of a constant dimensions of 4514.0 ± 17.6 pixels. For each mouse, DIA echodensity was obtained as the main value obtained from 4 frames of the same acquisition drawing the region of interest (ROI) in the same area of DIA muscle.

Both for GC and DIA, variations in echodensity were expressed as percentage differences between WT and *mdx* groups (treated or untreated). All data sets were obtained by repeated, independent analyses to avoid intra- and inter-variability, and multiple analyses consistency and reproducibility was ensured as previously described (38).

### 2.2 *Ex vivo* studies

At the end of the 8^th^ week of treatment, the *ex vivo* experimental phase started. Each mouse was anesthetized *via* intraperitoneal (i.p.) injection with a cocktail of ketamine (100 mg/kg) and xylazine (16 mg/kg). If required, an additional lower dose of ketamine alone (30 mg/kg) was injected for longer and deeper sedation. Both right and left hind limb tibialis anterior (TA), extensor digitorum longus (EDL), quadriceps (QUAD), GC, and SOL muscles were isolated and weighed. After heart removal, also DIA muscle was harvested from each animal. A section of right hemi-diaphragm and EDL muscle from the left limb were carefully prepared for contractile recordings (12, 32, 35). For all mice, GC muscle from the right limb was snap frozen in N_2_ and stored at −80°C until further processing for RT-PCR. In parallel, left hemi-diaphragm (costal muscle domain) and GC muscle of the left hind limb were embedded in a small amount of Tissue-Tek O.C.T. (Bio-Optica, Milan, Italy), immersed for 60 s in isopentane cooled with liquid nitrogen (N_2_), and then stored at −80°C until being further processed for histology. Both QUAD muscle of the left hind limb and the liver were snap frozen in N_2_ and stored at −80°C for further PK analysis. Blood was obtained *via* cardiac puncture by using an insulin syringe and a collection tube, both coated with a 2% solution of anticoagulant ethylenediaminetetraacetic acid (EDTA) disodium salt dihydrate (Sigma-Aldrich). Within 30 min after collection, platelet-poor plasma was obtained after two consequential centrifugation steps (20 min, 2000× g, 4°C; 10 min, 10,000× g, 4°C). An aliquot was used fresh to measure creatine kinase (CK) and lactate dehydrogenase (LDH) by spectrophotometry, whilst two more aliquots were stored at −80°C until pharmacokinetics (PK) and IGF-1 level measurement *via* enzyme-linked immunosorbent assay (ELISA) were performed. In addition, the remaining harvested hind limb muscles and organs were snap frozen in N_2_ and stored at −80°C in our mouse tissue repository for possible future analyses.

#### 2.2.1 Isometric contraction recordings of isolated muscles

A strip of right hemi-DIA (no more than 4 mm wide) and left EDL muscle were prepared to be placed each into a recording chamber containing 25 ml of isotonic Ringer’s solution (composition in mM: NaCl 148, KCl 4.5, CaCl_2_ 2.0/2.5, MgCl_2_ 1.0, NaH_2_PO_4_ 0.44, NaHCO_3_ 12.0, glucose 5.55; pH 7.2–7.4; gassed with 95% O_2_ and 5% CO_2_, at 27 ± 1°C) [TREAT-NMD SOP (ID) Number: DMD_M.1.2.002]. The DIA strip was placed into a vertical muscle bath, with the central tendon fixed to a hook at the bottom of the chamber, and the rib fixed to a dual-mode muscle lever (mod. 300C-LR, ASI); similarly, EDL muscle was placed into a horizontal muscle bath (mod. 809B-25, ASI), with the proximal tendon fixed to a 300C-LR force transducer and the distal tendon fixed to a hook at the opposite side of the chamber. Electrical field stimulation was obtained by two axial platinum electrodes closely flanking each muscle, connected to a high-power bi-phase stimulator (for DIA: LE 12406, 2Biological Instruments; for EDL: mod. 701C, ASI). Each apparatus was equipped with a data acquisition signal interface (for both systems: mod. 604A, ASI) and software (for DIA: DMCv4.1.6; for EDL: DMCv5.415, ASI). After equilibration (~30 min), muscle preparations were stretched to their optimal length (L0, measured with an external caliper), which is the length producing the maximal single contraction (twitch, Ptw) in response to a 0.2 ms square wave 40 – 60 mV pulse. Single twitch force and kinetics (Ptw; time to peak, TTP; half relaxation time, HRT) were obtained as mean values from 5 twitches elicited by pulses of 0.2 ms, every 30 s. Tetanic contractions were elicited by applying trains of 2.0 ms pulses for 450 ms (DIA) or 350 ms (EDL), at sequential increasing frequencies (from 10 to 250 Hz), every 2 min. Maximal tetanic force (P0) was usually recorded at 140–180 Hz. Data were analyzed *via* ASI software DMAv3.2 for DIA and DMAv5.201 for EDL, to obtain TTP, HRT, Ptw and P0 values, these latter then normalized to muscle cross sectional area according to the equation sP = P/(Mass/Lf*D) where P is absolute tension, Mass is the muscle mass, D is the density of skeletal muscle (1.06 g/cm^3^), Lf was obtained by multiplying L0 by previously determined muscle length to fiber length ratio (DIA = 1; EDL = 0.44). Hz50, a calcium-related parameter indicating the frequency at which 50% of maximal sP0 is produced, was also calculated from force-frequency curves (12, 32, 35).

#### 2.2.2 Muscle histology

Serial cross-sections (10 µm thick) from each frozen left DIA and GC muscles were transversally cut into a cryostat microtome set at −20°C (HM 525 NX, Thermo Fisher Scientific, Waltham, MA, USA) and slides (Superfrost™ Plus, Thermo Fisher Scientific) were stained. Muscles’ morphological features were identified using digital images, acquired with a bright-field microscope (CL-I Eclipse Nikon) and the image capture software ImageJ^®^. Classical histological hematoxylin and eosin staining (H&E; Bio-Optica) was used to estimate muscle architecture and to calculate the area of damage and regeneration (*i.e.* necrosis with presence of inflammatory infiltrates, fibrosis, regenerated tissue, altogether defined as unhealthy tissue; a sample image is provided as **Supplementary Figure 1**). Specifically, morphometric analysis was performed according to an internationally validated protocol (TREAT-NMD SOP (ID) Number: DMD_M.1.2.007), *via* a semi-automated process using the ImageJ^®^ software color deconvolution plugin (https://imagej.net/Deconvolution), which separates the hematoxylin component and the eosin component. Normal, undamaged myofibers are represented by the eosin component and this area can be measured by ImageJ^®^ automatic thresholding. Total tissue area is determined on the original picture.

Masson’s trichrome staining (Bio-Optica) was used for the detection of collagen (%) as an index of muscle fibrosis. The positive (blue) areas were quantified using ImageJ^®^ and normalized to the total area of single sections. The analyses were conducted on the entire muscle section, randomly selecting approximately 6 –10 fields per section at 10X magnification for muscle damage and 8 – 12 fields per section at 20X for collagen percentage (39).

#### 2.2.3 Isolation of total RNA, reverse transcription, and qRT-PCR

Total RNA was isolated from frozen GC muscles after homogenization, with Trizol (10296028, Life Technologies, CA, USA) and quantified for yield and purity by spectrophotometry (ND-1000 NanoDrop, Thermo Fisher Scientific). Variable amounts of RNA (between 1-2 µg) were then retro transcribed to cDNA with iScript gDNA Clear cDNA Synthesis Kit (172-5035 Bio-Rad Laboratories, Inc., CA, USA) following manufacturer instructions. qRT-PCR was performed *via* the CFX384 Connect Real-Time PCR System (Bio-Rad, Hercules, CA, USA) by using PrimePCR assays SYBR^®^ green with custom made plates. For each experiment, samples were analyzed with technical duplicates/triplicates. The mRNA expression of genes was normalized to the mean of two housekeeping genes, *β-actin* and Hypoxanthine phosphoribosyl transferase 1 (*HPRT1*) and quantified by the 2^ΔΔct^ method (40). Primers were purchased with the following Unique Assay IDs: β-actin (*Actb*), qMmuCED0027505; HPRT1 (*Hprt1*), qMmuCID0005679; collagen, type I, alpha 1 (*Col1a1*) qMmuCID0021007; sirtuin 1 (*Sirt-1*) qMmuCID0015511; PGC-1α (*Ppargc1a*) qMmuCID0006032; interleukin 6 (*IL-6*) qMmuCID0005613; Transforming growth Factor Beta (*TGF-β1*) qMmuCID0017320; Cluster of Differentiation 68 (*CD68*); qMmuCED0003822; cluster of differentiation 36 (*CD36*) qMmuCID0014852; Myogenin (*MYOG*) qMmuCED0001043; Myogenic Factor 5 (*MYF5*) qMmuCED0003779; Embryonic myosin heavy chain (*MYH3*) qMmuCID0022333; Myocyte Enhancer factor 2 (*MEF2C*) qMmuCID0005168; Insulin growth factor 1 (*IGF-1*) qMmuCED0044388.

#### 2.2.4 Biochemistry and pharmacokinetics

The enzymatic activity of CK and LDH in plasma samples (in U/L) was determined using specific commercially available diagnostic kits (CK NAC LR and LDH LR, SGM, Rome, Italy). Both the assays required the use of a spectrophotometer (Ultrospec 2100 Pro UV/Visible, Amersham Biosciences, Little Chalfont, United Kingdom) set to a wavelength of 340 nm at 37°C and were performed according to the manufacturer’s instructions (33, 34). IGF-1 levels were determined in plasma samples *via* commercially available ELISA, according to the manufacturer’s instructions.

The quantification of EP80317 and JMV2894 concentration was carried out in plasma, liver and QUAD samples from mice groups treated with both drugs in comparison to untreated *mdx* mice used as negative control. QUAD muscles and liver were weighed and homogenized in 1g/5ml with 20mM ammonium formate buffer with the Precellys homogenizer in 7ml vials with 7500 rpm speed 2cycles of 25sec each and 15 sec of pause. Samples were kept on ice before and after the homogenization to avoid degradation. Initial Stock solution was prepared in DMSO (2 mg/ml). Working solution were prepared as below. 5 µl of Working solutions (WS) were spiked in 45 µL of mouse plasma or tissue homogenates to obtain the desired final concentration. 50µl of samples and of calibrants were precipitated with 200 µl of cold ACN containing 10ng/ml/ml of Verapamil as internal standard (IS). Samples were shaken, centrifuged and supernatants were transferred into a 96 well plate and injected into a UPLC-MS/MS. Calibration range was from 1 to 2000ng/ml for all the three matrices for JMV2894 and 10ng/ml for EP80317 in all the three matrices. Samples were analyzed on a Acquity UPLC (Waters) coupled with a API3200 Triple Quadrupole ABSciex.

### 2.3 Molecular docking simulations and molecular interaction fields analysis

The two-dimensional X-ray structures of ADAMTS-5 and MMP-9 were retrieved from the Protein Data Bank (PDB entries 6YJM (41) and 1GKC (42), respectively). These target structures were refined by using the Protein Preparation Wizard (43) in order to correct bond order, add hydrogen atoms and possible missing aminoacidic side chains and loops. As elsewhere reported (42), two functional water molecules have been retained (that are HOH2038 and HOH2056 based on 1GKC numbering) within the MMP-9 binding site. The 3D conformation of the cognate ligands GLPG1972 for 6YJM and N~2~-[(2R)-2-{[formyl(hydroxy)amino]methyl}-4-methylpentanoyl]-N,3-dimethyl-L-valinamide (NRH) for 1GKC were processed by using LigPrep (44) in order to properly generate all the possible tautomers and ionization states at a physiological pH value of 7.4. A cubic grid centered on the cognate ligands GLPG1972 and NRH having an edge of 12 Å × 12 Å × 12 Å and 28 Å × 28 Å × 28 Å and 10 Å × 10 Å × 10 Å and 23 Å × 23 Å × 23 Å for the inner and outer boxes, have been automatically generated. Docking simulations were thus performed using Grid-based Ligand Docking with Energetics (GLIDE) v.9.1, which is part of the Schrodinger Suite (Schrödinger Suite 2021–4) (45). All the default settings for extra precision (XP) were used and the Force Field OPLS_2005 was employed. The reliability of the XP simulation protocol was validated by computing the Root Mean Square Deviations (RMSD) values of the cognate ligands. For the sake of completeness, all the molecular interactions observed in docking simulations are shown. To identify a broader spectrum of energetic contributions of the target residues, the BioGPS algorithm (46, 47), licensed by Molecular Discovery Ltd. (www.moldiscovery.com), was employed. BioGPS computed the molecular interaction fields (MIFs) to evaluate the type, strength, and direction of potential molecular interactions. Overall, the procedure implied the embedding of the target protein into a 3D grid centered first on each co-crystallized ligand (that are GLPG1972 and NRH for the ADAMTS-5 and MMP-9, respectively). Each considered region was thus processed by employing CRY, O, and N1 GRID probes to compute lipophilic, H-bond acceptor, and H-bond donor interactions, respectively. Once the MIFs were generated, each residue was associated with the type of generated interaction (CRY, N1, O) along with the corresponding energy value (expressed as kcal/mol).

### 2.4 Cell culture

Wild-type (WT) H2K-2B4 and dystrophic H2K-SF1 cell lines were cultured and used for all the experiments (48). Cells were seeded in 96-well cultures (approximately 4–4.5 × 10^3^ cells *per* well) and sixteen hours after seeding were treated with EP80317, JMV2894 (both in the concentration range 100μM to 1mM) or ghrelin (as control in the concentration range 10nM to 100 μM). Cell viability was then evaluated at different time points following the exposure (16-96 h) by using the Cell Counting Kit-8 (CCK-8,96992 Sigma-Aldrich), according to the instruction. The optical density of each well was determined using a microplate reader set to 450 nm (Victor 3V, Perkin Elmer, Waltham, MA, USA). Cell viability was expressed in percentage vs. control properly corrected for blank values.

### 2.5 Statistical analysis

All data were expressed as mean ± standard error of the mean (SEM). Multiple statistical comparisons between groups (WT, *mdx*, *mdx* + EP80317, *mdx* + JMV2894) were performed by *one-way* analysis of variance (ANOVA), with Bonferroni’s t-test *post hoc* correction when the null hypothesis was rejected (*p < 0.05*). All data followed, with good approximation, a normal distribution, being included in the 95% confidence interval of the mean. This generally allows to identify outliers, if any, and to apply the statistical analyses described above. No outliers were found, and the exclusion of specific samples from the analyses of the results was related only to overt technical issues during the experiments. *In vivo* and *ex vivo* procedures, data collection and analysis were conducted in a blinded fashion by the experimenters.

Whenever appropriate, the recovery score (RS), an objective index directly indicating how much of the deficit is recovered (%) by a treatment, was calculated according to TREAT-NMD (SOP (ID) Number: DMD_M.1.1.001), as follows:


$$\text{Recovery score}=\frac{\left(\text{treated }mdx\text{ mice}-\text{untreated }mdx\text{ mice}\right)\;}{\left(\text{control mice}-\text{untreated }mdx\text{ mice}\right)}\;x\;100$$

## 3 Results

### 3.1 Effects of GHSs administration on *in vivo* parameters

**Figure 1:**
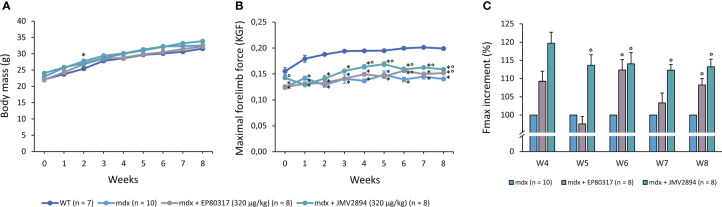
Values for body mass (BM, g; **A**) and maximal forelimb grip strength (KGF; **B**) at each weekly time point, and percentage of Fmax increment (%; **C**) from W4 to W8, for all mice cohorts. Values are expressed as mean ± SEM from the number (n) of mice indicated in brackets. A statistically significant difference among groups was found by one-way ANOVA for BM at T2 (F > 3; p < 0.05) and for Fmax (F > 10; p < 0.0001) and Fmax increment (F > 4.9, p < 0.01) at all time points. Bonferroni *post-hoc* test for individual differences between groups is as follows: * vs. WT (0.02 < p < 0.05); ° vs. *mdx* (0.0004 < p < 0.04).

The 8-week administration of EP80317 and JMV2894 did not modify the physiological age-related increase in BM, with an expected trend towards higher values in *mdx* vs. WT mice (**Figure 1A**).

Notably, maximal forelimb force, measured *via* grip strength meter, was significantly increased in treated *mdx* mice vs. untreated ones from week 4 onwards, particularly in those treated with JMV2894, with recovery scores ranging from 20 to 47% (**Figure 1B**); this was further highlighted by the quantification of the Fmax increment percentage (**Figure 1C**). This trend reoccurred when Fmax values were normalized to mice BM (**Supplementary Figure 2A**). On the contrary, no significant amelioration by either drug was found on hind limb plantar flexor torque in *mdx* mice (**Supplementary Figure 2B**).

**Figure 2:**
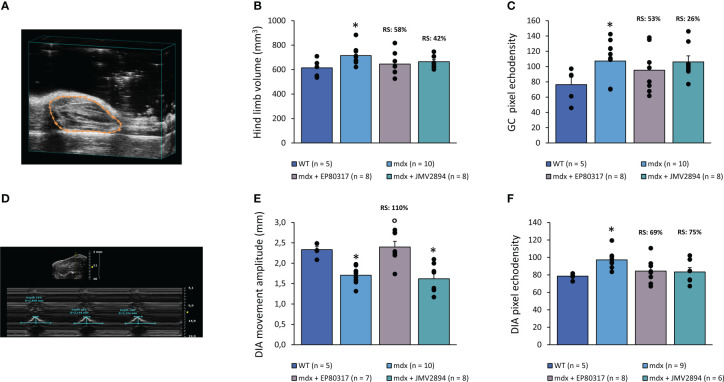
In **(A)** is shown a representative 2D image of ultrasound hind limb acquisition used to measure both volume and echodensity of gastrocnemius muscle (GC; highlighted in orange). Values for hind limb volume (mm^3^) and GC echodensity measured in all mice groups (WT, *mdx*, *mdx* treated with EP80317 or JMV2894) are shown in **(B, C)**, respectively, expressed as mean ± SEM from the number (n) of mice indicated in brackets. A statistically significant difference among groups was found by one-way ANOVA for B (F = 3.51, p < 0.03) and C (F = 3.05, p < 0.05). Bonferroni *post hoc* test for individual differences between groups is as follows: * vs. WT (0.03 < p < 0.05). In **(D)** is shown a representative image of B- (*upper panel*) and M- (*lower panel*) mode DIA acquisition allowing to measure diaphragm (DIA) echodensity and amplitude, respectively. Values for DIA movement amplitude (mm) and echodensity measured in all mice groups are shown in **(E, F)**, respectively, expressed as mean ± SEM from the number (n) of mice indicated in brackets. A statistically significant difference among groups was found by one-way ANOVA for E (F = 15.3, p < 0.0002) and F (F = 3.5, p < 0.03). Bonferroni *post-hoc* test is as follows: * vs. WT (0.0008 < p < 0.05); ° vs. *mdx* (p < 0.0002).

At T8 all animals underwent an ultrasonographic assessment of hind limb (**Figures 2A–C**) and DIA muscle (**Figures 2D–F**). The treatment with EP80317 or JMV2894 partially restored the increase in hind limb volume observed in untreated *mdx* mice (showing a significant +20.3% compared to WT), with a RS of 58% and 42%, respectively (**Figure 2B**). In untreated *mdx* mice, a significant increment in GC echodensity (+52.4%) was also observed, with EP80317 or JMV2894 partially ameliorating this parameter, with a RS of 53% and 26%, respectively (**Figure 2C**). Untreated *mdx* mice were characterized by a significant reduction of DIA amplitude (−73%) and a significant increase in echodensity (+24%) compared to WT mice. Interestingly, EP80317 significantly improved *mdx* DIA amplitude (RS: 110%) (**Figure 2E**). DIA echodensity of *mdx* mice was improved by EP80317 or JMV2894 treatment, with a RS of 69% and 75%, respectively (**Figure 2F**).

### 3.2 *Ex vivo* results

#### 3.2.1 Effects of GHSs on contractile parameters of isolated DIA and EDL muscles

**Figure 3:**
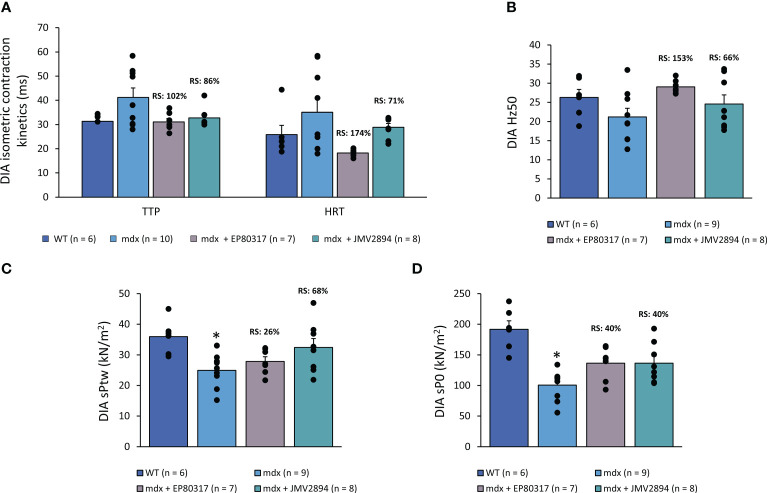
Isometric twitch contraction kinetics (time to peak, TTP, and half-relaxation time, HRT, ms; **A**), Hz50 (*i.e.* the frequency at which 50% of maximal isometric specific tetanic force is produced; **B**), maximal specific isometric twitch (sPtw, kN/m^2^; **C**) and tetanic (sP0, kN/m^2^; **D**) forces. Values are expressed as mean ± SEM from the number (n) of mice indicated in brackets. A statistically significant difference among groups was found by one-way ANOVA for TTP and HRT (F > 3.3, p < 0.04), sPtw (F = 4.7, p < 0.01), and sP0 (F = 11.6, p < 0.0001). Bonferroni *post-hoc* test for individual differences between groups is as follows: * vs. WT (0.001 < p < 0.05). Recovery scores (RS) toward WT values are indicated above the bars.

Data from isometric contraction recordings performed *ex vivo* in isolated DIA muscles from all mice cohorts are shown in **Figure 3**. Twitch kinetics were slower in untreated *mdx* mice compared to WT, with longer time-to-peak (TTP) and half relaxation time (HRT). The treatment with EP80317 or JMV2894 markedly improved both parameters, with high recovery scores (**Figure 3A**). In parallel, untreated *mdx* DIA showed a lower Hz50 compared to WT. This index, together with TTP and HRT may be related to altered Ca^2+^ homeostasis characterizing dystrophic muscles. Interestingly, both compounds recovered Hz50 (RS: 153% for EP80317, 66% for JMV2894) corroborating a potential ability to ameliorate Ca^2+^ handling (**Figure 3B**). As expected, untreated *mdx* mice exhibited a drastic, statistically significant reduction in both sPtw (**Figure 3C**) and sP0 (**Figure 3D**) compared to WT mice. Both force indices were remarkably improved by GHSs. In contrast, no remarkable effects of either GHS were observed on isolated EDL muscle, with a modest, if any, recover of the significantly impaired sPtw and sP0 (**Supplementary Figures 3A, B**).

#### 3.2.2 Effects of GHSs on histopathology of GC and DIA muscles

**Figure 4:**
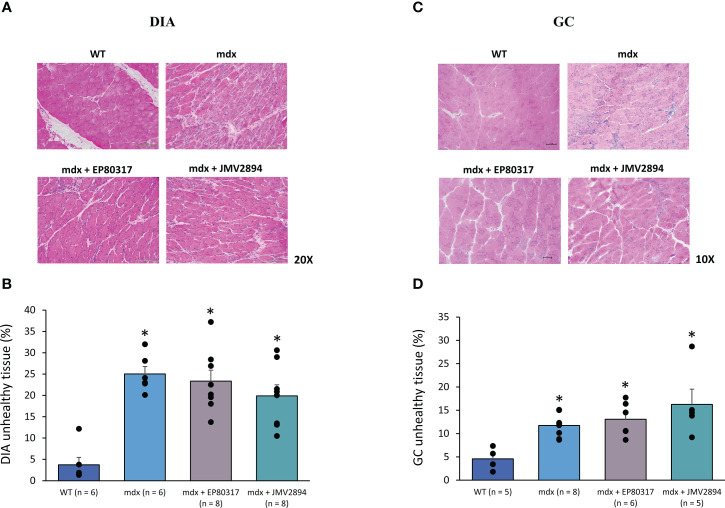
In **(A, C)** are shown representative DIA (20× magnification) and GC (10× magnification) muscle sections stained with H&E from mice of each experimental group. This staining allows to appreciate the areas of unhealthy tissue, quantified *via* subsequent morphometric analysis, as shown by graphs in **(B)** for DIA and **(D)** for GC muscles. Values are expressed as mean ± SEM from the number of mice (n) indicated in brackets. A statistically significant difference among groups was found by one-way ANOVA for DIA (F = 15.1 p < 0.0001) and GC (F = 6.9, p < 0.002). Bonferroni *post-hoc* test for individual differences between groups is as follows: DIA: * vs. WT (0.0001 < p < 0.0004); GC: * vs. WT (0.02 <p < 0.03).

The histopathology in DIA and GC muscle sections was evaluated by H&E staining (**Figures 4A–D**). The typical hallmarks of dystrophic histopathology – altered muscle architecture with extensive areas of unhealthy tissue, comprising areas in active necrosis with inflammatory infiltrates, or occupied by non-muscle tissue – were evident in both muscles (**Figures 4A, C**), with higher percentages of unhealthy tissue in *mdx* DIA with respect to GC muscle (**Figures 4B, D**). A slight amelioration of DIA histopathology was found in treated *mdx* mice, whereas no protection was exerted on GC muscle, exhibiting a trend towards increased mean values in treated groups, possibly related to inter-individual variability.

**Figure 5:**
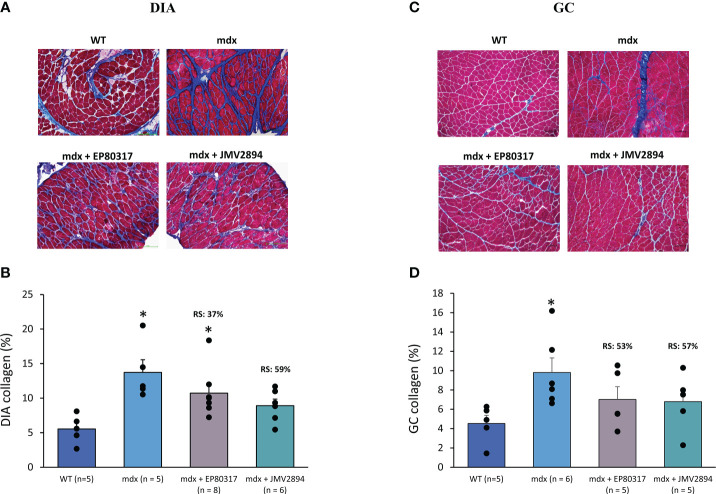
In **(A, C)** are shown representative DIA (20× magnification) and GC (10x magnification) muscle sections stained with Masson’s Trichrome from mice of each experimental group. This staining allows to appreciate the collagen deposition (in blue), quantified *via* subsequent morphometric analysis, as shown by graphs in **(B)** for DIA and **(D)** for GC muscles. Values are expressed as mean ± SEM from the number (n) of mice indicated in brackets. A statistically significant difference among groups was found by one-way ANOVA for DIA (F = 6.3, p < 0.003) and GC (F = 3.4, p < 0.04). Bonferroni *post-hoc* test for individual differences between groups is as follows: DIA: * vs. WT (0.003 < p < 0.04); GC: * vs. WT (p < 0.03).

The effects of both EP80317 and JMV2894 administration on fibrosis levels of DIA and GC muscles was assessed by Masson’s Trichrome staining. Representative pictures of muscle sections are shown in **Figures 5A, C**). Collagen content was significantly increased in *mdx* mice, with values twice higher than those observed in both DIA and GC WT muscles (**Figures 5B, D**). Both GHSs, especially JMV2894, were able to partially reduce the extent of collagen deposition in both muscles, with RS ranging from 37 to 59%.

#### 3.2.3 Modulation of gene expression by EP80317 and JMV2894 in GC muscle

**Figure 6:**
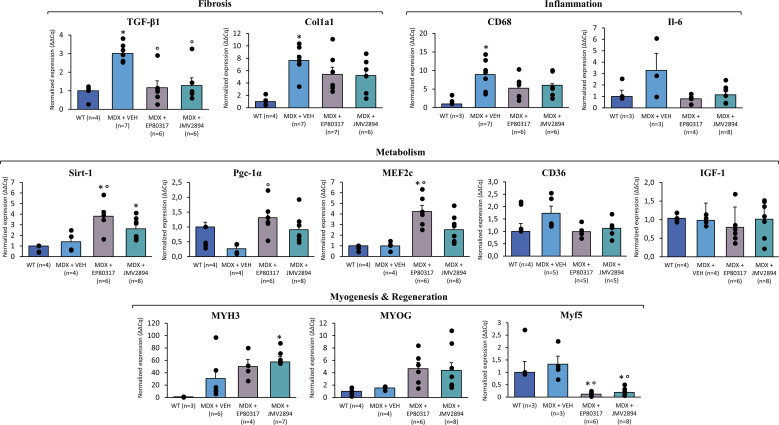
Transcriptional levels, measured by qRT-PCR in GC muscle, of genes related to fibrosis (*TGF-β1; Col1a1*), inflammation (*CD68; IL-6*), muscle metabolism (*Sirt-1; Pgc-1α; MEF2c; CD36; IGF-1*), myogenesis and regeneration (*MYH3; MYOG; Myf5*). Values are expressed as mean relative quantity ± SEM from 4 to 10 samples per group, normalized to the mean of housekeeping genes *β-actin, HPRT1*. A statistically significant difference was found among mice groups by one-way ANOVA for *TGF-β1, Col1a1, CD68, Sirt-1, Pgc-1α, MEF2c, MYH3, Myf5* (F > 19; p < 0.0001). Bonferroni *post hoc* test for individual differences between groups is as follows: * vs. WT (0.0001 < p < 0.06); ° vs. *mdx* (0.0001 < p < 0.01). No significant differences were found for *IL-6, MYOG, CD36, IGF-1*.

**Figure 6** shows the results obtained from gene expression experiments in GC muscle. As expected, the expression levels of genes involved in pro-fibrotic pathways, such as *Col1a1* and *TGF-β1*, were significantly higher in *mdx* compared to WT mice. Interestingly, both genes were downregulated in treated *mdx* mice (in a statistically significant manner for *TGF-β1*), in line with an anti-fibrotic effect of the two compounds. In parallel, the expression levels of inflammation markers *CD68* and *IL-6*, were higher in *mdx* mice compared with WT, and markedly reduced by both treatments.

GC muscles from mice treated with both GHSs, and particularly with EP80317, showed an increased expression of *Sirt-1* and *PGC-1α*. Notably, these results paralleled the increase in *MEF2c* expression, supporting a general stimulation of the oxidative metabolism and regulation of mitochondrial biogenesis. Moreover, the expression of *CD36*, the main scavenger responsible of the internalization of fatty acids and collagen inside cells, was slightly upregulated in *mdx* mice vs. WT and reduced by both treatments.

Genes responsible for the myogenic cascade such as *Myf5* and *Myogenin* were respectively reduced and increased in both EP80317 and JMV2894 treated mice compared to WT and untreated *mdx*, opening the hypothesis that the two GHSs were able to interfere with myogenic program. On the other hand, dystrophic muscles treated with both compounds showed a further trend toward increment of *Myh3* (embryonic myosin heavy chain) gene levels vs. untreated *mdx* mice, which suggests a pro-regenerative effects of GHSs.

Interestingly, and according to previous studies (49), there was no expression of *GHSR-1a* gene (encoding for Growth Hormone Secretagogue Receptor) in all muscles (data not shown). In addition, no relevant effect of GHSs administration on *IGF-1* expression was detected (**Figure 6**).

#### 3.2.4 Disease biomarkers and IGF-1 levels in plasma and pharmacokinetic analysis

**Figure 7:**
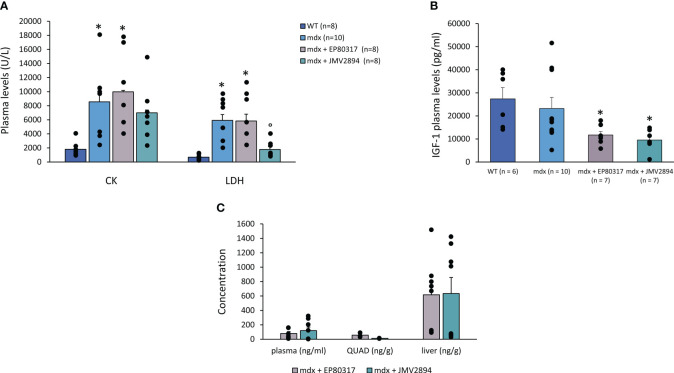
In **(A)** are shown the levels of enzymes creatine kinase (CK) and lactate dehydrogenase (LDH) (U/L), measured in plasma samples collected from mice of each experimental group. Values are expressed as mean ± SEM from the number (n) of mice indicated in brackets. A statistically significant difference among groups was found by one-way ANOVA for both CK (F = 6.4, p = 0.002) and LDH (F = 13.4, p = 8.10*10^-6^). Bonferroni *post hoc* test for individual differences between groups is as follows, CK: * vs. WT (0.0003 < p < 0.01); LDH: * vs WT (4.23*10^-5^ < p < 1.6*10^-5^), ° vs *mdx* (0.0002 < p < 0.0004). In **(B)** are shown the levels of IGF-1 measured in plasma samples collected from mice of each experimental group (pg/ml). A statistically significant difference among groups was found by one-way ANOVA (F = 5.9, p = 0.004). Bonferroni *post hoc* test for individual differences between groups is as follows: * vs. WT (0.004 < p < 0.02). In **(C)** is shown the concentration of EP80317 and JMV2894 in plasma (ng/ml), quadriceps muscle (QUAD), and liver samples, carried out in n = 8 samples for each type of matrix. A total of n = 2-4 samples of plasma and muscle tissue have been excluded from each group after the measurements, since their values were below the level of quantification (LOQ). Data are expressed as mean ± SEM. Vehicle samples (not shown) always resulted below the LOQ for both GHSs, excluding any possibility of contamination. No significant difference was found by one-way ANOVA followed by Bonferroni *post-hoc* test.

As expected, plasma levels (U/L) of CK and LDH enzymes resulted significantly higher in dystrophic animals. JMV2894 induced a decrease in plasma levels of each enzyme, which resulted statistically significant vs. untreated mice for LDH. Conversely, no effects were detected after EP80317 administration for either enzyme (**Figure 7A**).

In parallel, IGF-1 plasma levels were reduced by both drugs, suggesting a GH-independent direct effect of the two compounds on skeletal muscle (**Figure 7B**).

Results from *ex vivo* PK analysis to measure the traceability of each GHS in treated *mdx* mice plasma (ng/ml), QUAD muscle, and liver (ng/mg) are shown in **Figure 7C**. Both EP80317 and JMV2894 were found predominantly in the liver, and in lower amounts in plasma and QUAD muscle samples from treated *mdx* mice. During the measurements, variability was noted among the subjects, although generally mice with higher plasma exposure also showed a higher content in liver and QUAD muscle.

### 3.3 Docking and molecular modeling of interaction with ADAMTS-5 and MMP-9

Based on the claimed ability of GHS to inhibit the metalloprotease ACE enzyme (30) an *in silico* assessment was carried out for JMV2894 on two metalloproteases involved in extracellular matrix re-modelling and inflammatory signaling, namely ADAMTS-5 and MMP-9. To corroborate the validity of docking studies, redocking simulations have been first performed on the co-crystallized ligands in their binding site. Cognate ligands moved back to their original positions with Root Mean Square Deviations (RMSD) values, accounting for all the heavy atoms, as small as 1.083 Å and 0.638 Å for GLPG1972 on ADAMTS-5 and NRH on MMP-9, respectively (**Supplementary Figure 4**).

**Figure 8:**
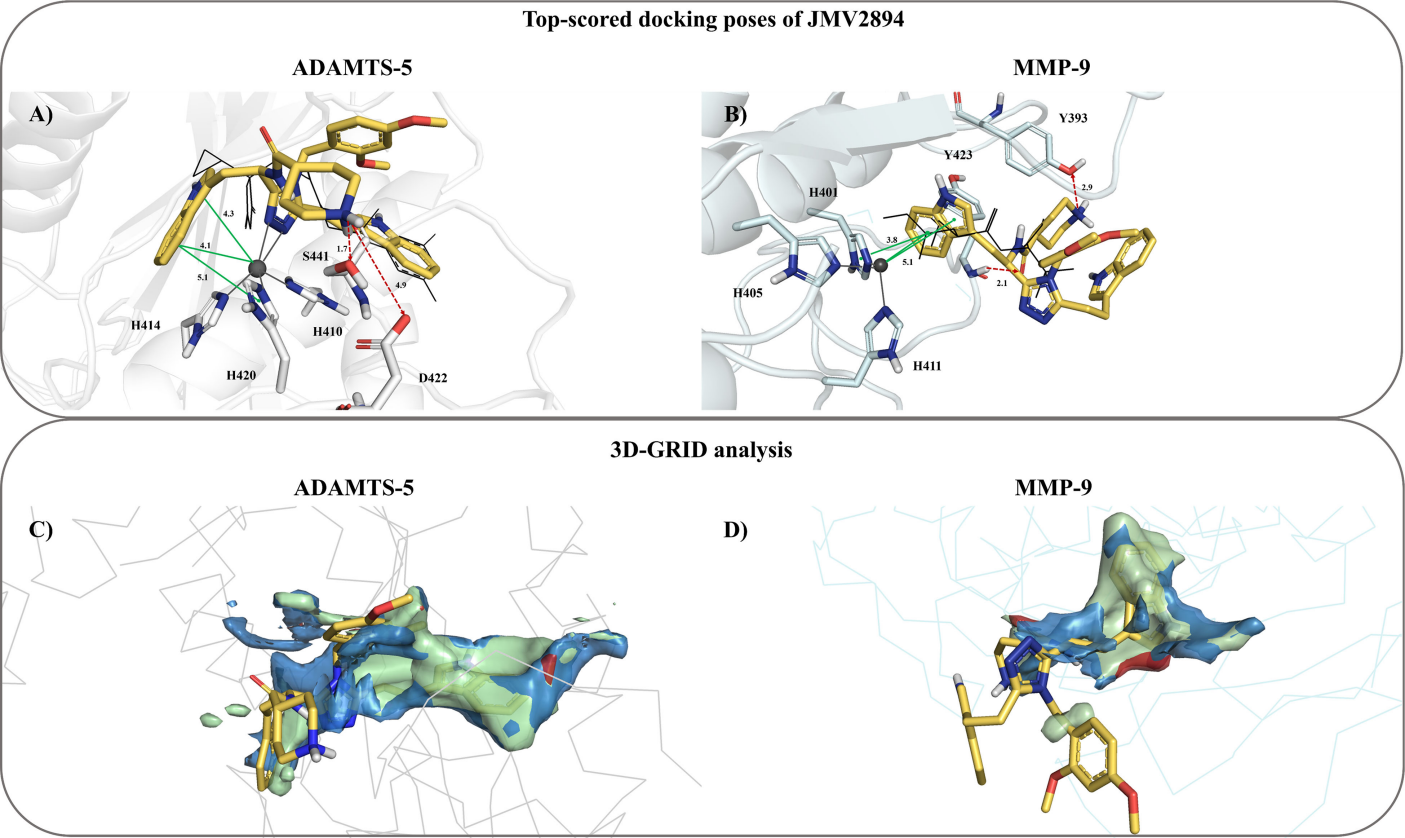
Top-scored docking poses of JMV2894 towards **(A)** ADAMTS-5 (PDB entry: 6YJM) and **(B)** MMP-9 (PDB entry: 1GKC), respectively. Green and red arrows indicate π − π or π -cationinteractions and hydrogen bonds, respectively. ADAMTS-5 and MMP-9 binding site residues arerendered in grey and cyan sticks, respectively. For the sake of completeness, all molecularinteractions observed in docking simulations are shown. GRID MIFs generated on the basis of **(C)** ADAMTS-5 and **(D)** MMP-9 crystal structures. The hydrophobic, HB acceptor, and HB donorinteractions are displayed in green, red, and blue surface, respectively. A cut-off value of –1.0kcal/mol, −4.5 kcal/mol, and −3.0 kcal/mol is set for CRY-, N1-, and O-GRID probes, respectively. The top-scored docking poses of JMV2894 are reported as yellow sticks, while ADAMTS-5 andMMP-9 binding sites are rendered in grey and cyan ribbon, respectively.

Extra precision docking simulations were thus exploited to investigate the binding modes of JMV2894 compound towards ADAMTS-5 and MMP-9 crystal structures. All the docking scores are summarized in **Table 1** whereas both top-scored docking poses as well as the relevant binding residue interactions are depicted in **Figures 8A, B**. Interestingly, JMV2894 returned a docking score which was slightly higher than that of GLPG1972, the co-crystallized ADAMTS-5 ligand. On the contrary, JMV2894 returned a docking score resulting slightly lower than that of NRH, the co-crystallized MMP-9 ligand. Noteworthy, docking poses of JMV2894 showed a substantial overlap with both the co-crystallized inhibitors.

**Table 1:** Docking score of JMV2894 towards ADAMTS-5 and MMP-9 crystal structures expressed as kcal/mol.

	Docking score (kcal/mol)	
	ADAMTS-5	MMP-9
JMV2894	-10.198	-5.950
GLPG1972	-7.740	–
NRH	–	-8.931

For the sake of completeness, all values for the cognate ligands are also reported.

As elsewhere reported (41, 42), the Zn^2+^ ion was coordinated with the imidazole groups of three catalytic histidines (i.e., H414, H420 and H410 for ADAMTS-5 and H405, H411 and H401 for MMP-9). The obtained docking poses of JMV2894 showed its 1,2,4 triazole ring establishing a putative bidentate coordination with the Zn^2+^ ion of ADAMTS-5 and its indole group making π-cation contacts with the Zn^2+^ ions of ADAMTS-5 as well as of MMP-9. Additionally, two π-π contacts were returned with the H420 of ADAMTS-5 and H401 of MMP-9 at a distance of 5.1 Å and 3.8 Å, respectively. As far as the molecular interactions were concerned, the JMV2894 was able to engage hydrogen bonds with the S441 of ADAMTS-5 and Y423 and Y393 of MMP-9. The piperidine moiety also established salt bridge with D422 of ADAMTS-5.

An *in silico* investigation of the ADAMTS-5 and MMP-9 binding sites was also carried out by analyzing the molecular interaction fields (MIFs) generated by each X-ray crystal structures by using BioGPS algorithm (46, 47) (**Figures 8C, D**). MIFs generated through the GRID force field using three GRID probes (i.e., CRY, N1, and O) of the BioGPS algorithm were employed to assess lipophilic, H-bond acceptor, and donor interactions, respectively. The width of a given MIF depended on the energy of the interacting groups involved. This implied that the larger the MIF, the higher was the chance to find a complementary partner group in that molecular region (50). For the hydrophobic residue pairs, both S1’ pocket binding residues interacted with the hydrophobic CRY probe by producing a large MIF (dark green surface isocontours), which intercepted a region with a high possibility to detect hydrophobic groups (that were the indole groups of JMV2894 in both binding sites of ADAMTS-5 and MMP-9 crystal structures). Different observations hold true for the polar interactions. For instance, the N1 probe interacting with the two metalloproteinases cavities residues generated MIFs (blue surface isocontours) indicating a high probability to detect a H-bond donor groups. This was less experienced by top-scored docking poses of JMV2894 excepting for the nitrogen of indole groups.

Overall, the docking simulations integrated with GRID analysis allow us to hypothesize that JMV2894 can adopt two different and effective binding modes towards ADAMTS-5 and MMP-9.

### 3.4 *In vitro* evaluation of potential cytotoxic effects of selected GHSs

To assess any potential EP80317 and JMV2894 toxicity on muscle precursors that can minimize the effect on regeneration, both compounds were tested *in vitro* on two different murine immortalized satellite-derived cell lines, the WT H2K-2B4 and the dystrophic H2K-SF1. Toxic effect was evaluated both in proliferation and differentiation conditions. Ghrelin was used as control. No toxic effect was exhibited at any EP80317 and JMV2894 tested concentration in proliferating or differentiating 2B4 or SF1 cells. Also, no toxic effect was exerted by ghrelin at 1 mM in both conditions (**Supplementary Figures 5A–D**).

## 4 Discussion

The potential use of GHSs as a pharmacological strategy in DMD might represent an exclusive therapeutic opportunity, considering their wide mechanisms of action and their reported ability to contrast metabolic dysfunctions and damaging signals in wasting muscle conditions such as sarcopenia and chemotherapy-related muscle cachexia (27–29). Nonetheless, the development of robust preclinical data concerning the actual efficacy profile of these compounds in DMD settings is still in its infancy. In this frame, the present work aimed to expand the current knowledge about GHSs pharmacodynamics, through a multidisciplinary approach that allowed to analyze the effects of GHSs administration on several pathology-relevant aspects in the commonly used murine model of DMD, the *mdx* mouse.

The two tested compounds, EP80317 and JMV2894, were used at a dose proven to be effective in other experimental conditions (30) and did not exhibit any cytotoxic effects *in vitro*, nor caused remarkable changes in body weight of treated mice. Both drugs were able to improve forelimb strength *in vivo*, with a significant effect exerted by JMV2894. Conversely, this result was not supported by plantar flexor torque measures. Both techniques are internationally recognized as reliable measures of muscle function, and the apparent discrepancy can be easily reconciled considering the different nature of each measurement, *e.g.* the different muscle groups involved, the concerted function of vascular and nervous systems, and animal behavior in non-anesthetized mice (12).

Interestingly, GHSs also ameliorated the function of DIA muscle, a result of particular relevance considering that this muscle more closely reproduces the alteration observed in human dystrophic conditions. Both compounds contrasted the drastic impairment of *ex vivo* contractile force parameters (sPtw, sP0) characterizing *mdx* mice, meanwhile restoring contraction kinetics and reducing Hz50 values. These alterations are likely related to the impaired calcium homeostasis of dystrophic myofibers and to the “leaky” Ryanodine Receptor 1 (RyR1), possibly due to the effect of oxidative stress and related aberrant nitrosylation (32). The GHSs can exert a stabilizing action on RyR1 and store-operated calcium entry (SOCE mechanism), also described to be altered in dystrophic fibers, by a direct effect on these structures (28) or by reducing oxidative stress. Importantly, the amelioration of DIA function in response to each treatment was also highlighted *in vivo* by ultrasonography experiments.

By contrast, the minor effects observed *in vivo* and *ex vivo* on hind limb function may be due to either a limited exposure to the drug or to a different threshold sensitivity of different muscular district, also in relation to the different damage extent affecting DIA and EDL muscle.

By the way, collagen deposition, a hallmark of dystrophic muscles in both patients and *mdx* mice, represents a pathway of interest for GHSs action. In fact, Masson’s trichrome staining highlighted a marked reduction of collagen percentage in DIA and GC muscles of treated animals, supporting the anti-fibrotic effect of GHSs. This is in line with previous studies conducted in animal models of cardiac hypertrophy and acute lung injury (51, 52), demonstrating GHSs’ ability to reduce collagen deposition and to attenuate fibrosis. Further evidence about the anti-fibrotic activity of GHSs treatment was also provided by ultrasonography, and particularly by reduced echodensity of DIA and GC muscles in treated *mdx* mice. Despite the lack of a direct mathematical correlation between the techniques, due to clear methodological differences, we previously demonstrated that a higher echodensity value is strictly related to (and in line with) an increased collagen deposition or to fatty tissue replacement in muscle myofibers and may also account for impaired function (38). The accumulation of fibrotic tissue indeed reduces muscles elastic properties, compromising diaphragmatic movements during the inspiration/expiration process, providing another mechanism for enhanced DIA function *in vivo* and *ex vivo*. Furthermore, *mdx* mice exhibited a lower hind limb volume when treated with each GHS. Importantly, it must be considered that dystrophic muscles are routinely heavier and larger vs. healthy ones, precisely in relation to the pseudohypertrophy triggered by the fibro-fatty replacement (53). Given this, the lower hind limb volume observed in GHSs treated animals could be associated with the potential ability of these drugs to control this compensatory hypertrophy and, again, with the decrease of fibrotic/adipose tissue. Importantly, in treated *mdx* mice, we observed a marked downregulation of key genes related to fibrosis and inflammation, while the upregulation of the SIRT-1/PGC-1α pathway and the increased expression of *MEF2c*, suggest a parallel improvement of mitochondrial function and oxidative capacity triggered by GHSs treatment. Although the expression of metabolism-related genes may be subjected to fluctuations in relation to disease phase and activity (11), it is worth underlining that an GHSs enhancement of these pathways was observed in other muscle wasting conditions (29). The stimulation of *Sirt-1* expression might also be involved in the reduction of fibrosis, in view of the ability of this deacetylase of reducing TGF-β1 activity (54). Treated *mdx* mice showed a marked downregulation of *Myf5* and higher levels of *myogenin*, suggesting that GHSs had the ability to stimulate myoblast differentiation, likely through the enhancement of MyoD signaling pathway. Furthermore, we detected an upregulation of *Myh3* after GHSs administration, confirming the pro-regenerative potential of these compounds. This may deserve dedicated experiments to verify direct GHS action on myogenesis of dystrophic muscle precursors also in view of regenerative medicine approaches. A downregulation of the *CD36* gene, the most important fatty acid transporter in skeletal muscle was also observed. This event might contribute to reduce the lipids overload in dystrophic myofibers, hypothesis further strengthened by the evidence that EP80317 is a ligand of CD36 translocase (55).

Given the beneficial effects observed concerning the reduction of fibrosis and the recovery of muscle strength, we wondered about the exact mechanism through which GHSs exerted their action. According to literature (49), gene expression analysis did not reveal the presence of detectable levels of the GHS-R1a in GC muscles. In addition, IGF-1 plasma levels were paradoxically reduced in GHSs treated mice. This may result from a possible dual agonist/antagonistic action of both compounds on pituitary GHS-R1a in condition of normal ghrelin production (26). However, the data prove that the observed effects are independent of GH-IGF-1 axis. The existence of a strong correlation between the progression of fibrosis and the aberrant extracellular matrix (ECM) remodeling in dystrophic myofibers, due to the production of pro-inflammatory and pro-fibrotic signaling molecules, led us to investigate other potential sites for drug action. In particular, we hypothesized that ADAMTS-5 and MMP-9, two metalloproteinases, overexpressed in DMD patients and in *mdx* mice (56, 57), might be potential novel targets of GHSs in skeletal muscle, based on the observation that both compounds may indeed act as inhibitors of other metalloproteinases, such ACE. It is known that ADAMTS-5 blockade with a specific monoclonal antibody reduced the extent of fibrotic tissue and ameliorated dystrophic pathology in *mdx* mice, as well as the administration of batimastat, a competitive inhibitor of MMPs (57, 58). To date, it recently emerged that ghrelin and hexarelin are able to downregulate MMP-9 and ADAMTS-5 expression and suppress their activity *via* the inhibition of pro-inflammatory cytokines like IL-1, TGF-β1 and TNF-α, all metalloproteinases’ stimulators (59, 60). In detail, we performed docking studies to verify the binding mode between JMV2894, the GHS compound with a small-molecule-like structure, and both metalloproteinases. Overall, the docking simulations integrated with GRID analysis disclosed that JMV2894 could adopt two different and effective binding modes towards ADAMTS-5 and MMP-9. Notably, the docking score of JMV2894 was higher than that of GLPG1972, a selective ADAMTS-5 inhibitor, corroborating the hypothesis that a direct interaction between JMV2894 and the enzyme might occur. This paves the way to future dedicated biological experiments.

In conclusion, the present preclinical study highlighted, for the first time, the potential benefits and tolerability resulting from the GHSs’ administration in dystrophic conditions. Forthcoming studies will be aimed to test the antifibrotic activity of GHSs in a late disease stage, when an exacerbation of fibrosis occurs, or in a novel murine model of DMD, the D2.B10-Dmd*^mdx^*/J mouse, characterized by a more severe pro-fibrotic phenotype. This model, which shows an earlier cardiomyopathy, would also allow to verify the potential ability of GHS to contrast the heart dysfunction and remodeling, a key aspect in pathology severity. Also, an insight into novel formulation and delivery systems may help to improve GHSs bioavailability and to obtain a greater muscle exposure, so to predict greater target-tissue effects on pathological processes.

Importantly, further experiments will be required to confirm the biological outcome of the direct interaction between ghrelin mimetics and fibrosis-related metalloproteinases also in view of the therapeutic potential of these molecules in DMD and other disorders characterized by excessive fibrosis and pathological tissue remodeling.

## Data availability statement

The original contributions presented in the study are included in the article/**Supplementary Material**, further inquiries can be directed to the corresponding author/s.

## Ethics statement

The animal study was reviewed and approved by National Ethics Committee for Research Animal Welfare of the Italian Ministry of Health (authorization no.166/2020-PR).

## Author contributions

ADL, AL, BB, OC, and PM were responsible for the conception and design of the *in vivo* / *ex vivo* study. BB, OC, PM, AM, and FS carried out *in vivo* experiments. BB, OC, PM, LT, MDB, SC, FS, AGC, EC, RM, LR, and EB all contributed to *ex vivo* and *in vitro* experiments at their sites. SD and J-AF synthetized the compounds. ON, DT, and GC were responsible for the molecular docking study. ADL, BB, OC, and PM drafted the manuscript; BB, PM and DT prepared, assembled, and revised figures and tables. ADL, PM, BB, OC, J-AF, AL, and AT contributed to revise the final version and the revision of the manuscript.
