# Supplementary material for: Growth hormone secretagogues modulate inflammation and fibrosis in mdx mouse model of Duchenne muscular dystrophy
Source: Front Immunol. 2023 Apr 12;14:1119888. doi: 10.3389/fimmu.2023.1119888 (PMC10130389; doi:10.3389/fimmu.2023.1119888)
Supplement: Supplementary file 1 [file DataSheet_1.pdf]

Suppl. Figure 1

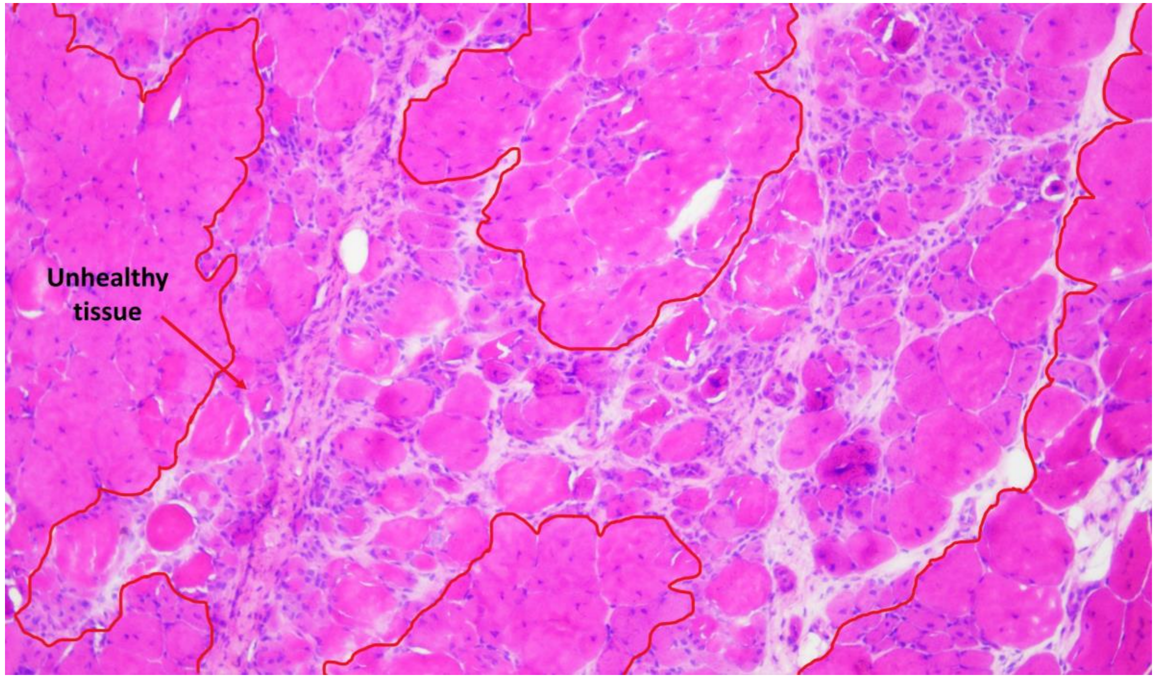

Suppl. Figure 1. Sample image of one *mdx* gastrocnemius (GC) muscle section (10× magnification), stained with H&E. Unhealthy tissue, comprising areas in active necrosis with inflammatory infiltrates, non-muscle tissue (fibrosis and fatty connective tissue), and regenerated areas (myofibers with central nuclei), is outlined in red.

Suppl. Figure 2

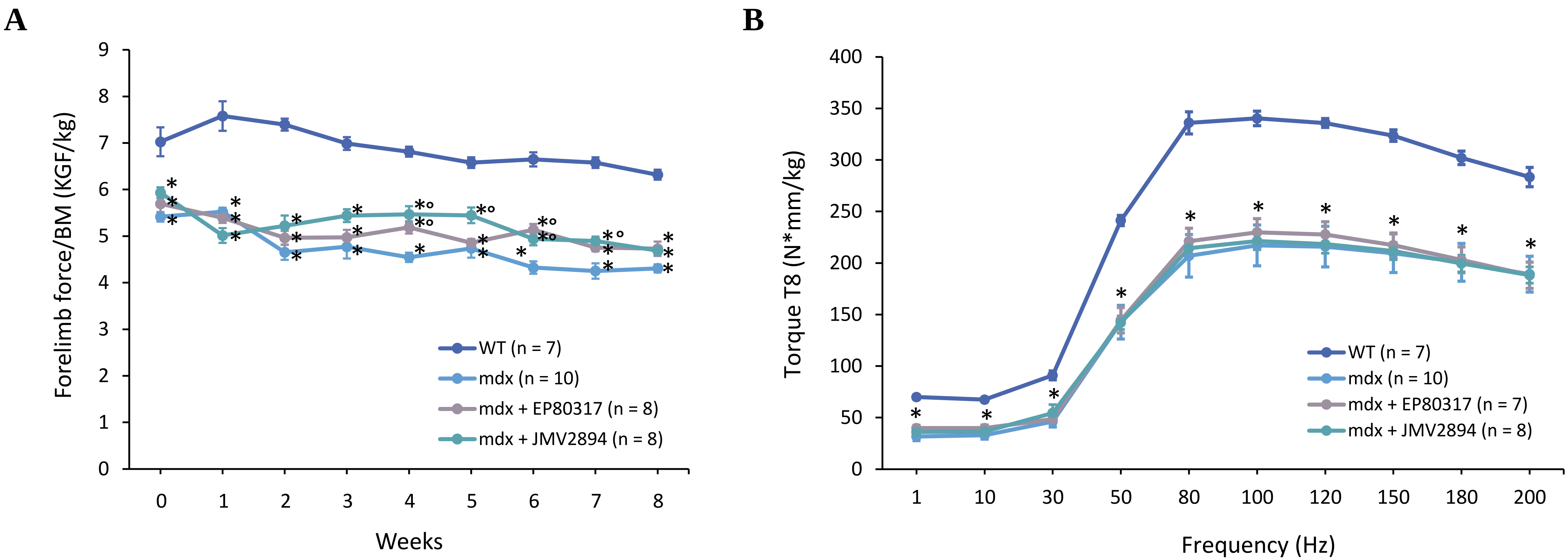

Suppl. Figure 2. A) Values of forelimb grip strength normalized to body mass (BM; KGF/kg) at all weekly time points for all mice cohorts. Values are expressed as mean ± SEM from the number of mice indicated in brackets. A statistically significant difference among groups was found by one-way ANOVA ($F > 15$, $p > 0.0001$). Bonferroni post hoc test for individual differences among groups is as follows: * vs. WT (p < 0.05); ° vs. *mdx* (p < 0.05). **B)** Hind limb plantar flexor torque (N*mm/kg) produced at increasing stimulation frequencies (from 1 to 200 Hz), obtained in anesthetized mice from each cohort at T8. Values are expressed as mean ± SEM from the number of mice indicated in brackets. A statistically significant difference among groups was found by one-way ANOVA at T8 ($F > 11.7$, $p < 0.0001$) at all stimulation frequencies. Bonferroni post hoc test for individual differences among groups is as follows: vs. *WT (0.001 < p < 0.05).

Suppl. Figure 3

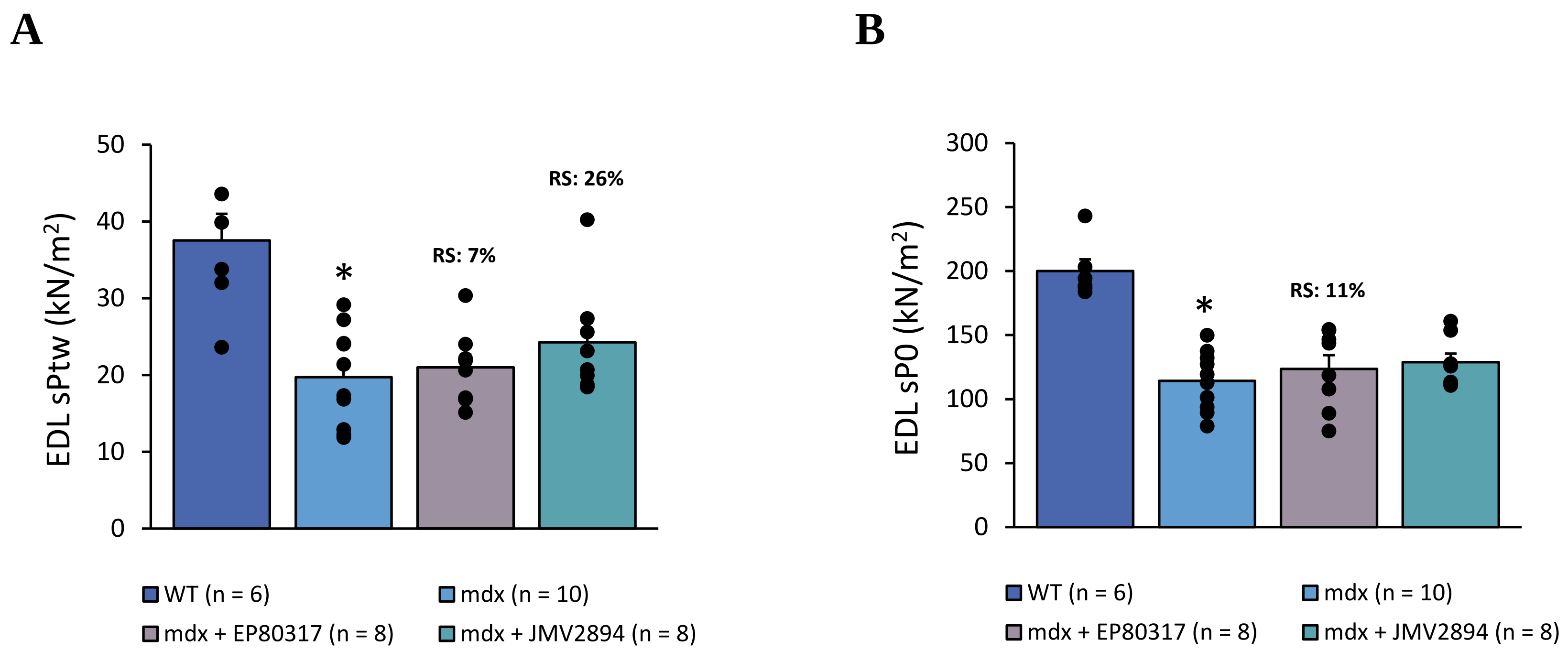

Suppl. Figure 3. In (**A**–**B**) are shown *ex vivo* maximal specific isometric twitch (sPtw, kN/m^2; **A**) and tetanic (sP0, kN/m^2; **B**) force values, obtained in EDL muscle for all mice cohorts. Values are expressed as mean ± SEM from the number of mice indicated in brackets. A statistically significant difference among groups was found by one-way ANOVA for both sPtw ($F = 8.5$, $p = 0.0004$) and sP0 ($F = 18.1$, $p < 0.0001$). Bonferroni post hoc test for individual differences between groups is as follows: * vs. WT ($0.001 < p < 0.05$). Recovery scores (RS) toward WT values are indicated above the bars.

Suppl. Figure 4

Redocking simulations

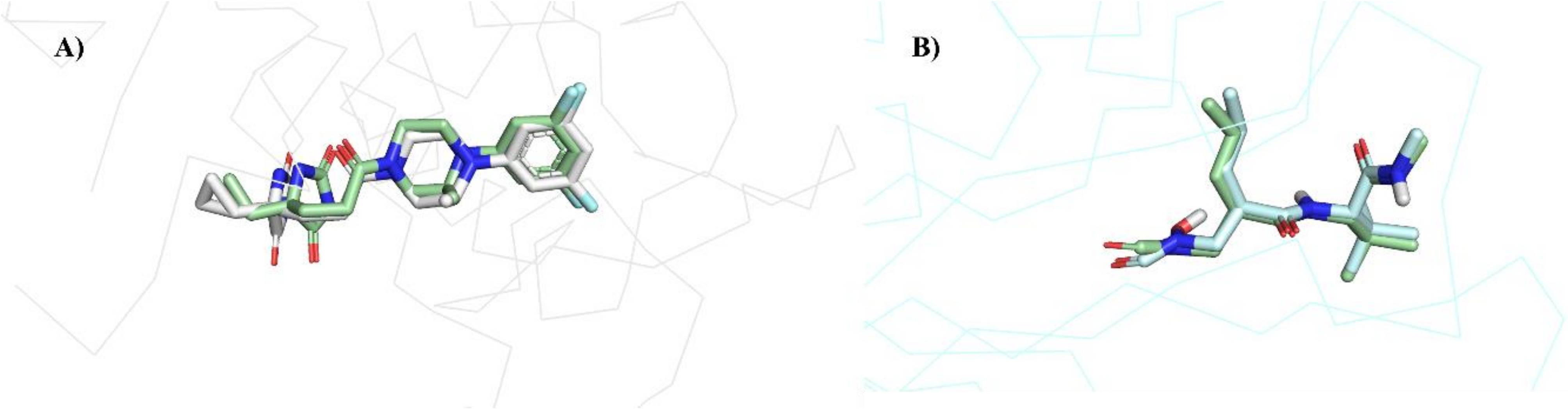

Suppl. Figure 4. Overlap of X-ray solved (green sticks) and docking pose of cognate ligand of A) GLPG1972 and B) NRH in grey and cyan sticks, respectively. The crystal structures of ADAMTS-5 and MMP9 are depicted in grey and cyan ribbon, respectively.

Suppl. Figure 5

A

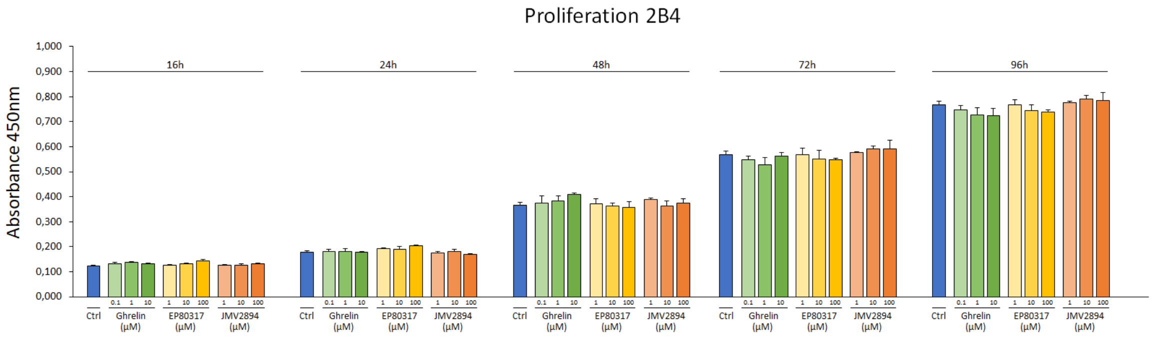

B

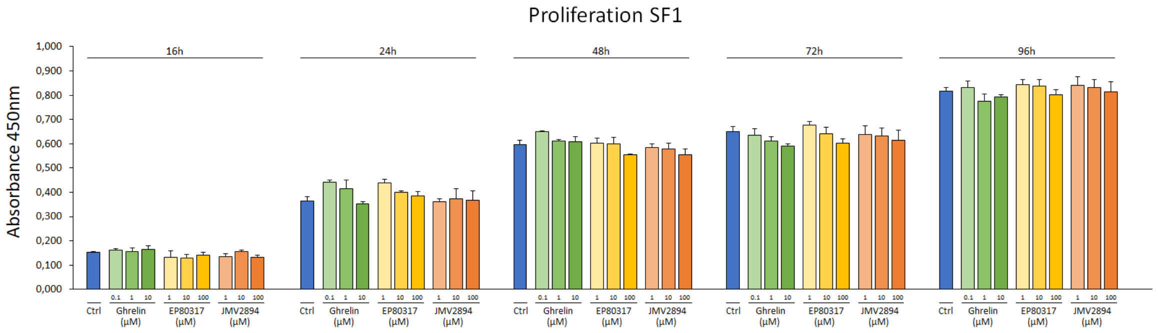

C

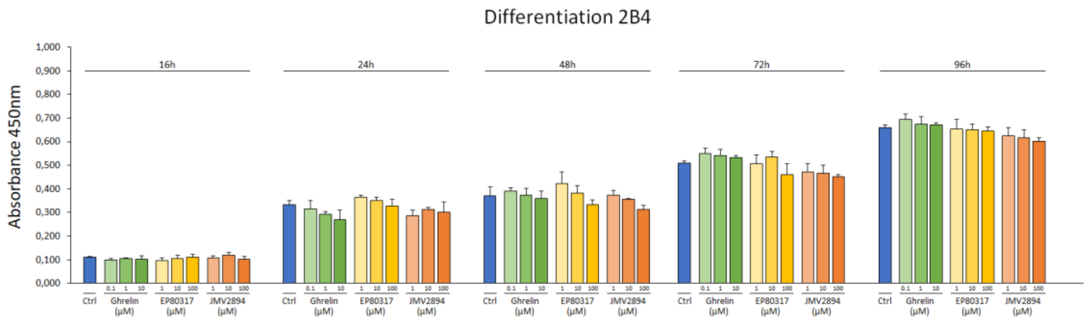

D

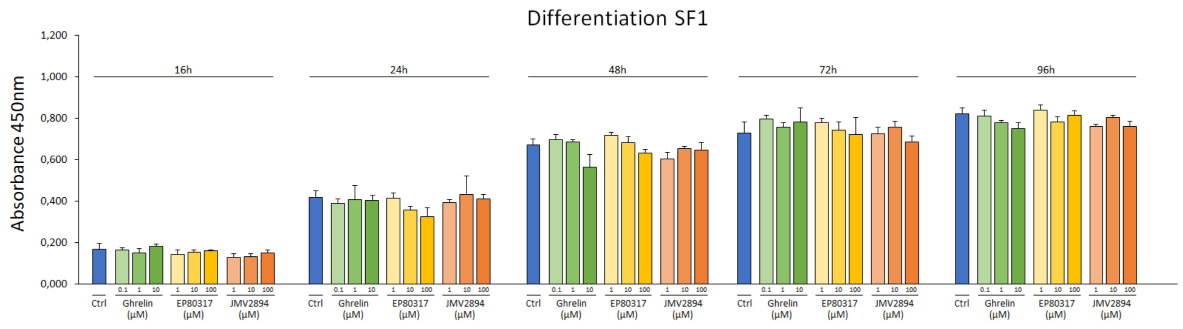

Suppl. Figure 5. *In vitro* assays to assess the potential cytotoxic effect of EP80317 and JMV2894 in H2K-2B4and SF1 muscle cell-lines. Experiments were performed in triplicate; values are expressed as mean ± SEM; CCK-8 was added to the cells 1h before reading at 450nm. Ctrl = 2B4/ SF1cells. No statistical significance was found between Ctrl and the three concentrations chosen for each compound (ghrelin, EP80317, or JMV2894) by one-way ANOVA.
